# Geniac: Automatic Configuration GENerator and Installer for nextflow pipelines

**DOI:** 10.12688/openreseurope.13861.2

**Published:** 2022-02-21

**Authors:** Fabrice Allain, Julien Roméjon, Philippe La Rosa, Frédéric Jarlier, Nicolas Servant, Philippe Hupé

**Affiliations:** 1 Mines Paris Tech, Fontainebleau, F-77305, France; 2 Institut Curie, Paris, F-75005, France; 3 U900, Inserm, Paris, F-75005, France; 4 PSL Research University, Paris, F-75005, France; 5 UMR144, CNRS, Paris, F-75005, France

**Keywords:** workflow management systems, containerization, reproducibility, high-performance computing, bioinformatics pipelines

## Abstract

With the advent of high-throughput biotechnological platforms and their ever-growing capacity, life science has turned into a digitized, computational and data-intensive discipline. As a consequence, standard analysis with a bioinformatics pipeline in the context of routine production has become a challenge such that the data can be processed in real-time and delivered to the end-users as fast as possible. The usage of workflow management systems along with packaging systems and containerization technologies offer an opportunity to tackle this challenge. While very powerful, they can be used and combined in many multiple ways which may differ from one developer to another. Therefore, promoting the homogeneity of the workflow implementation requires guidelines and protocols which detail how the source code of the bioinformatics pipeline should be written and organized to ensure its usability, maintainability, interoperability, sustainability, portability, reproducibility, scalability and efficiency. Capitalizing on Nextflow, Conda, Docker, Singularity and the nf-core initiative, we propose a set of best practices along the development life cycle of the bioinformatics pipeline and deployment for production operations which target different expert communities including i) the bioinformaticians and statisticians ii) the software engineers and iii) the data managers and core facility engineers. We implemented Geniac (Automatic Configuration GENerator and Installer for nextflow pipelines) which consists of a toolbox with three components: i) a technical documentation available at https://geniac.readthedocs.io to detail coding guidelines for the bioinformatics pipeline with Nextflow, ii) a command line interface with a linter to check that the code respects the guidelines, and iii) an add-on to generate configuration files, build the containers and deploy the pipeline. The Geniac toolbox aims at the harmonization of development practices across developers and automation of the generation of configuration files and containers by parsing the source code of the Nextflow pipeline.

## Introduction

With the advent of high-throughput biotechnological platforms and their ever-growing capacity, life science has turned into a digitized, computational and data-intensive discipline. While genomics was the major driving force for high-throughput sequencing, other fields including proteomics, imaging and microscopy now contribute to this data explosion ( Goh & Wong, 2020). Therefore, the bottleneck shifted from data generation to the ability to process and analyze data with efficient algorithms.

The data analysis actually encompasses two levels, corresponding to different time scales. The first level consists of the iterative *research development* of innovative and state-of-the-art analysis methods to address novel scientific questions. This level usually takes several months or years. In contrast, the second level consists of routine *production* analysis using mature and validated methods to apply standard analysis (including quality control) to the new samples processed by these high-throughput biotechnological platforms ( Reiter *et al.,* 2021). The *production* analysis must be performed within a short-delay (few hours up to few days) in order to process in real-time the huge data flow produced by these platforms, such that the relevant biological information can be extracted and provided to the end-users as fast as possible for their downstream analysis. This time constraint is even more important when the data are used in healthcare to support therapeutic decisions: in this case, quick parallel algorithms are required to process high-throughput sequencing data ( Jarlier *et al.,* 2020). Both levels feed each other in a virtuous circle.

Whatever the level, the analysis is generally a complex workflow (or pipeline) which involves many steps with different informatics languages and software, whether developed in-house or by third-parties. Coordinating the execution and allowing the scalability of these different steps to benefit from high-performance computing infrastructures to speed-up computation have been simplified with the development of workflow management systems ( Leipzig, 2017; Strozzi *et al.,* 2019) in combination with several technologies ( Gruening *et al.,* 2018; Tanjo *et al.,* 2021) including package management systems with Conda or Bioconda ( Grüning *et al.,* 2018), and containers with Docker ( Merkel, 2014) or Singularity ( Kurtzer *et al.,* 2017). Importantly, the use of workflow management systems also promotes reusable, reproducible and shareable analysis according to the FAIR principles ( Wilkinson *et al.,* 2016) which not only apply to data but also to workflows ( Goble *et al.,* 2020). Among the workflow management systems, Nextflow ( Di Tommaso *et al.,* 2017) became very popular and offers many interesting features ( Jackson *et al.,* 2021). It uses a dataflow programming model which implicitly defines the dependencies between the processes via their outputs and inputs such that different processes can be run in parallel and/or wait for each other when needed.

Whenever run for *production* analysis, the workflow has no choice but to succeed such that the results can be delivered in time to the end-users. If the workflow fails because of hardware issues or edge/corner cases due to the data themselves, then debugging, fixing, re-installing and resuming the workflow must be performed as fast as possible. This obviously requires harmonized guidelines and protocols across the different developers involved in the development life cycle of the pipeline. This way, it is easier to maintain many different pipelines on one hand and it simplifies the skill transfer from one person to another on the other hand. We developed the biogitflow protocols which cover the code versioning using git and GitLab ( Kamoun *et al.,* 2020). However, these protocols do not address how to write the code itself. While very valuable guidelines have been proposed to avoid pitfalls in the implementation of bioinformatics pipelines using the technological stack with workflows management systems, package management systems and containers ( Gruening *et al.,* 2018; Reiter *et al.,* 2021; Tanjo *et al.,* 2021), they remain very generic. Therefore, software engineering best practices and technical protocols for coding are necessary in order to promote software quality in bioinformatics to ensure the usability, maintainability, interoperability, sustainability, portability, reproducibility, scalability and efficiency of the workflows, as highlighted by several authors ( da Veiga Leprevost *et al.,* 2014; Georgeson *et al.,* 2019; Lawlor & Walsh, 2015).

We acknowledge the nf-core initiative ( Ewels *et al.,* 2020) which plays a major role in promoting the software engineering best practices to implement bioinformatics pipelines with Nextflow including code template, linter, code reviewing and continuous integration for better quality. Our Bioinformatics Core Facility decided to capitalize on both Nextflow and the nf-core initiative to provide the end-users with bioinformatics pipelines in the context of *production* analysis. In this article, we describe additional guidelines on how to write the Nextflow code. We propose a set of best practices along the development life cycle of the pipeline and deployment for production operations which address different expert communities: i) the bioinformaticians and statisticians who prototype the pipeline with state-of-the-art methods in order to extract most of the hidden value from the data and provide the end-users with summary reports, ii) the software engineers who optimize the pipeline to reduce the amount of required informatics resources, to shorten the time to result delivery, iii) the data managers and core facility engineers who deploy and operate the pipeline for daily *production* analysis for the end-users. The guidelines were motivated by: i) allowing the different expert communities to still work with their preferred work habits, ii) reducing the overall development cycle from the prototyping stage to deployment in a production environment, iii) providing portable pipelines with containers (Docker and Singularity), and iv) automating (whenever possible) the building of containers from the source code of the pipeline. Therefore, we implemented *Geniac* (Automatic Configuration GENerator and Installer for nextflow pipelines) ( Hupé *et al.,* 2022) to address these challenges. Geniac may be used alone for any pipeline built with Nextflow or as an extension of the nf-core guidelines to reduce the code complexity during the prototyping stage and standardize the deployment process.

Geniac actually consists of a toolbox with three components: a technical **documentation** available at https://geniac.readthedocs.io, a **command line interface** (CLI) with a linter to check that the code respects the guidelines, and an **add-on** to generate configuration files, build the containers and deploy the pipeline. We introduce here the main features of the Geniac toolbox.

## Methods

### General principle

The ultimate goal of the proposed best practices of coding with Nextflow was to ensure the portability and reproducibility of the pipeline with containerization techniques (such as Docker and Singularity). Therefore, the rationale behind Geniac was motivated by the automation of the construction of the containers without explicitly writing the Dockerfile or the Singularity Definition File recipes (whenever possible). To do so, the pipeline source code is parsed by Geniac . The portability of the pipeline with containers can be achieved in two ways: either a single container including all the tools required by the pipeline is provided, or several containers (one container for each tool to be as modular as possible) are provided. We decided to retain this second way, as this *one container - one tool* strategy was strongly recommended by several experts ( Gruening *et al.,* 2018) who stated that *Each container should encapsulate only one piece of software that performs a unique task with a well-defined goal (e.g. sequence aligner, mass spectra identification)*. Moreover, the *one container - one tool* strategy has the following advantages:

- most of the pipelines share tools in common meaning that, once a container is built for one tool, it can be easily reused in other pipelines,
- sometimes, different tools that might be incompatible with each other are required, making the usage of a single container including all the tools for the pipeline impossible,
- building a container with all the tools can be very long and possibly tedious. Each time the pipeline changes, the single container has to be rebuilt. With the *one container - one tool* strategy, only the container in which the tool has changed has to be updated (which is faster),
- the building of the containers can be parallelized to speed-up the deployment of containers.

Generally, the building of the containers occurs in the very late stage of the development cycle of the pipeline, once it has been validated by the end-users and is ready to be deployed in production. Therefore, the guidelines also took into consideration that the pipeline could simply be run during its very early stage of prototyping by bioinformaticians and statisticians, without systematically building the containers whenever a new version of the tool is tested or a new tool is added. This challenge can be easily tackled with the Conda packaging system ( Gruening *et al.,* 2018) which offers great flexibility to bioinformaticians and statisticians in order to install and test new tools (when available in the different Conda channels, which is the case for most of the bioinformatics tools). For this reason, the Geniac toolbox allows for the possibility of running the pipeline with different Nextflow profiles (a profile is a set of configuration attributes which can be activated/chosen when launching a pipeline execution by using the -profile command line option with Nextflow) including:

singularity to use the Singularity containers.

docker to use the Docker containers.

conda to use a single Conda environment on which all the tools are available.

multiconda to use a dedicated Conda environment for each tool.

path to define the PATH environment variable for which all the tools are assumed to be installed in the same directory.

multipath to define a dedicated PATH environment variable for which each tool is installed individually.

In addition to these profiles which set where the tools are accessible, we defined the cluster profile which can be combined with the previous profiles so that Nextflow launches the computation on a high-performance computing cluster.

As the Geniac toolbox automatically generates the configuration files used by these different profiles from the source code, it ensures that they are consistent with each other. Moreover, it saves time for the developer who does not have to write them one by one. All these different profiles are made available with the Geniac toolbox so that it covers all of the preferred work habits of a developer. However, we strongly encourage the use of multiconda during prototyping and singularity for production. Of note, both conda and path profiles may not work if the pipeline needs tools that are not compatible with each other.

**Figure 1.**
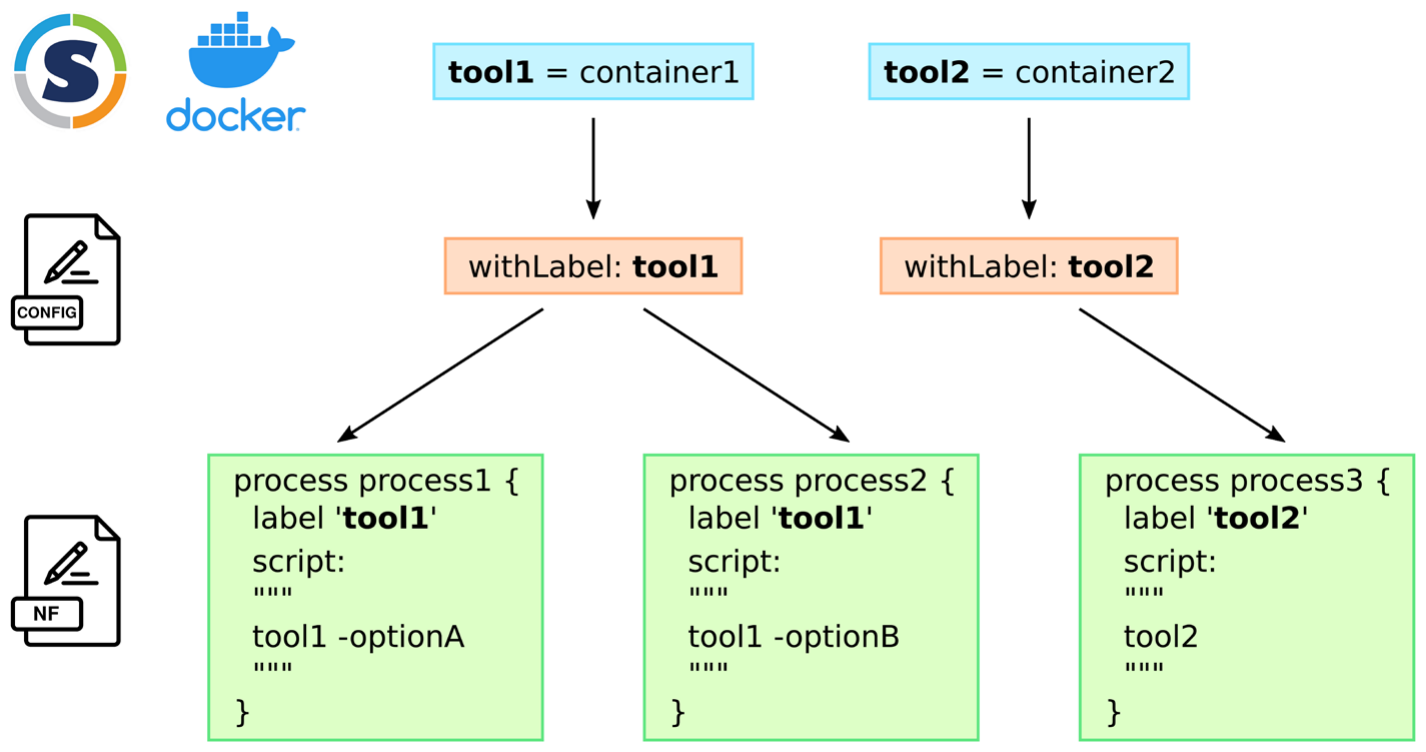
*one container - one tool* strategy and coding of the Nextflow pipeline: each tool that is used by the pipeline has its own container. In the different configuration files which are specific to a Nextflow profile, each tool is declared with the withLabel process selector. To use the appropriate tool in a Nextflow process, the use of the label directive with the name of the tool makes the link. The same tool can be used in different processes when different tasks have to be performed at different stages or with different options.

The *one container - one tool* strategy has to be translated in terms of coding best practices with Nextflow so that the source code can be automatically parsed to generate these Nextflow configuration files and build the containers. The coding relies mainly on the use of the label directive for each process which uses only one tool and one container at a time, along with the withLabel process selector defined in each configuration file for the different profiles which can be run with the -profile Nextflow option. Figure 1 illustrates this principle on two tools and three Nextflow processes.

### Availability of the tool

**Figure 2.**
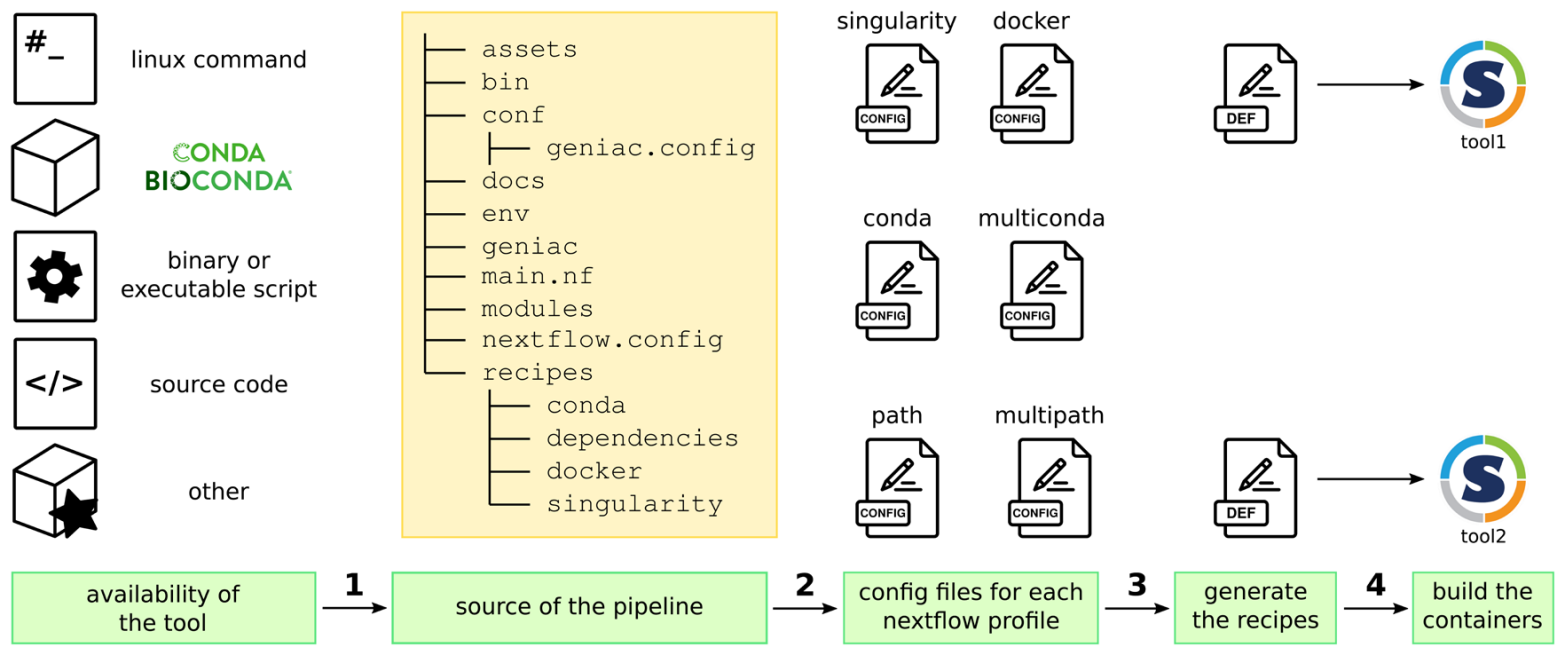
General principle of the Geniac guidelines and toolbox: 1/ a new tool is added in the pipeline according to the guidelines in the Geniac documentation depending on where the tool is available, 2/ the toolbox parses the structure of the source repository and the content of the conf/geniac.config file in order to automatically generate all the configuration files which define the Nextflow profiles, 3/ during the parsing, it also generates the container recipes (here the Singularity Definition Files) which are used to 4/ build the containers (here the Singularity images).

When implementing a new tool in a pipeline, the main question to answer is *Where is the tool available?* Depending on the answer, the developer has to follow specific guidelines which are fully detailed in the Geniac documentation. Whenever possible, it is recommended to use the tool from the Conda packaging system ( Gruening *et al.,* 2018). However, not all tools are available in Conda. Therefore, we guide the developer through a series of chronological questions which redirect the developer to the appropriate section whenever an answer is *Yes*. The questions are the following: i) *Is it just a standard Unix command?*, ii) *Is it available in Conda?*, iii) *Is it available only as a binary or as an executable script?*, iv) *Is the source code available?*, v) *Have you still not answered yes?* Briefly, the developer has to perform some actions such as modify the conf/geniac.config file, copy the source code of the tool in the modules/fromSource folder, write some recipes in the folder recipes, copy some files in the bin or recipes/dependencies folders, depending on the answer to the questions. In most of the cases, the tool should be available in Conda which is the most comfortable situation as it allows the automation of the creation of the containers and the configuration files for Nextflow profiles. However, the toolbox is able to tackle any other situations, although this requires more configuration on the developer’s side. Figure 2 describes the overall workflow with Geniac . 

### Implementation

**Figure 3.**
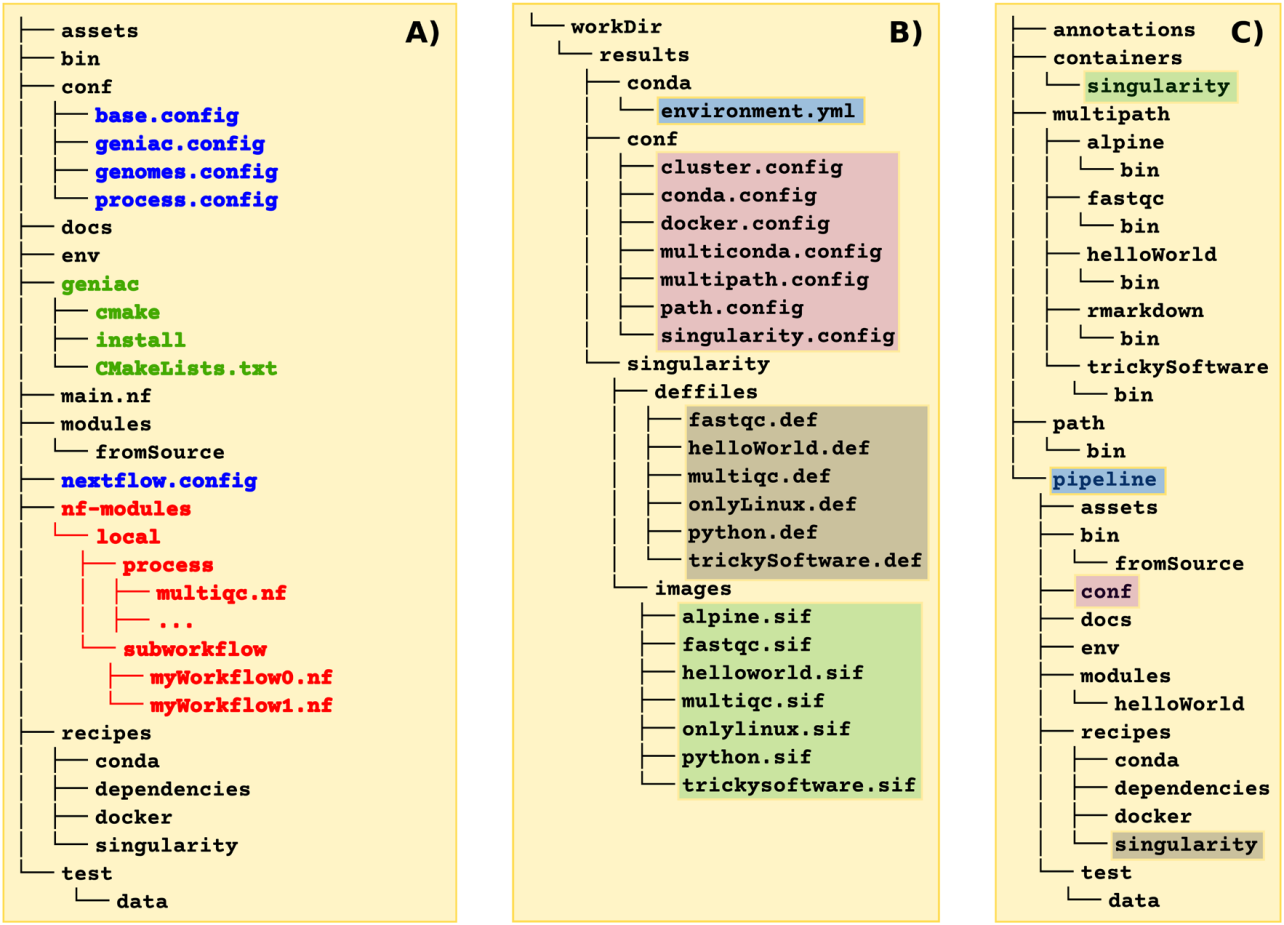
Organization of the different directories. **A) source code directory:** in blue are listed the mandatory files which could be retrieved from the Geniac Template , in green appears the folder of the Geniac toolbox itself which contains the files from the Geniac repository. Examples of source code are available on the GitHub repositories for both the Geniac Demo and the Geniac Demo DSL2 (in red is highlighted what is specific to a Nextflow pipeline which is implemented with DSL2). **B) build directory:** once the pipeline has been configured with cmake and built with make, a workDir folder appears with all the files which are automatically generated by Geniac . **C) install directory:** during the installation with make install several folders are created such as the pipeline folder which contains the Nextflow code. The files located within the same block in the build directory are copied in the folder of the same color in the install directory.

It is important to note that the Geniac toolbox ( Hupé *et al.,* 2022a) relies on the structure of the Geniac Template ( Servant & Hupé, 2022) while the latter could work without Geniac (provided that the missing configuration files for the different Nextflow profiles are added manually). It also means that the Geniac documentation explains how the Geniac Template works with the Geniac toolbox. Therefore, the source code must be organized as shown in Figure 3A with some mandatory files from the Geniac Template in blue and the Geniac toolbox itself in the eponymous folder in green. Based on this organization, the automatic generation of the Nextflow configuration files, container recipes and images are performed by some Nextflow scripts ( geniac/install/singularity.nf and geniac/install/docker.nf) which are invoked by Cmake scripts. The file nextflow.config includes all the configuration files that are automatically generated by Geniac to define the different Nextflow profiles. As it will be illustrated in the *Use Cases* section, the file gconf/geniac.config is essential as it allows registration of the tools available from Conda.The main Cmake script is the file geniac/CMakeLists.txt which invokes a series of functions from the geniac/cmake folder. The command line interface (CLI) corresponds to a standalone python package within the geniac/src folder. It includes a linter which offers the possibility for the developer to check the code’s compliance with the guidelines. The linter includes the following tests: i) existence of mandatory or optional files and folders in the Nextflow project tree structure, ii) configuration and consistency of labels with process directives in the Nextflow pipeline, iii) validation of the geniac configuration file, iv) availability of tools and their dependencies. Similarly to the nf-core linter, the output can be exploited manually or automatically within a continuous integration pipeline since any critical error reported by the linter changes the exit code from 0 to 1. The CLI also provides other commands for users not familiar with cmake or make commands to initiate a working directory, install and test the pipeline.

In order to ensure the reproducibility with Singularity, the geniac/install/singularity.nf script creates the singularity.config file such that Singularity is executed with specific options. Indeed, the default behavior of Singularity makes it possible to access the user’s $HOME directory from the host on which the process is executed. Some programming languages such as python or R preemptively load libraries from the user $HOME if they exist rather than the libraries from the canonical installation. To tackle this major issue, the singularity.config file i) sets the option "-B \"\$PWD\":/tmp --containall" which is passed on the Singularity command line when invoked by Nextflow and ii) sets "singularity.autoMounts = false" such that the developer explicitly decides during the deployment what folders have to be available within the container. The --containall sets some directories as empty (including $HOME and /tmp) thus avoiding possible libraries installed in the user’s $HOME to be loaded by these programming language. The -B option allowing the binding of the /tmp (inside the container) to the work directory of the Nextflow process (i.e. in the folder like work/2d/8202294 created by Nextflow during the execution) is also necessary. Indeed, the default overlay size of /tmp inside the Singularity image is few MB that makes the writing of temporary files required by many tools impossible due to space limitation. We preferred to bind /tmp to the work directory of the Nextflow process instead of on the actual /tmp of the host since it might use some RAM (tmpfs) which is not reported to the job scheduler (such as SLURM or PBS). Our configuration isolates the $HOME from the host whatever the programming language used inside a Nextflow process which makes this solution universal. However, this solves this issue only for the singularity profile. In the case of the conda or multiconda profiles, we addressed this issue by adding *ad-hoc* environment variables using the beforeScript directive beforeScript = "export R_LIBS_USER=\"-\"; export R_PROFILE_USER=\"-\"; export R_ENVIRON_USER=\"-\"; export PYTHONNOUSERSITE=1" in the files which are automatically generated by Geniac . If any other programming language suffers from the same behavior, *ad-hoc* solutions will have to be added. Note that the default options when using Docker containers within Nextflow avoid access to $HOME, this profile is therefore not concerned by the reproducibility issue.

### Operation

The Geniac toolbox is intended to run on a Linux distribution with Nextflow (>= 21.10.6), git (>= 2.0), Cmake (>= 3.0), Make (>= 4.1) and Conda (>= 4.10.1). To use the containers, either Apptainer/Singularity (>= 3.8.5) or Docker (>= 18.0) are needed. The Geniac command line interface requires python (>= 3.10).

## Use cases

In this section, several use cases are described. They are available in the bash script data/useCases.bash from the Geniac repository so that the reader can reproduce them step-by-step. The use cases depend on each other meaning that they must be sequentially performed from the beginning. They also require the installation of Nextflow (>= 20.01.0), git (>= 2.0), Cmake (>= 3.0), Make (>= 4.1), Conda (>= 4.10.1) and Singularity. The conducting line of this section is the deployment of a pipeline to be run in routine *production* with Singularity on a high-performance computing cluster having SLURM as a job-scheduler. A comprehensive overview of the Geniac functionalities is available in the Geniac documentation. 

### Create the geniac conda environment

In order to run the different use cases, create the geniac conda environment which provides all the dependencies needed:


export GENIAC_CONDA="https://raw.githubusercontent.com/bioinfo-pf-curie/geniac/release/environment.yml"

wget ${GENIAC_CONDA}
conda env create -f environment.yml
conda activate geniac


### Add a Nextflow process for a tool available in Conda

We explain here how to add a new process in the pipeline when the tool is available in Conda. First, the source code from the Geniac Demo ( Hupé *et al*., 2022b) repository can be downloaded as follows:


export WORK_DIR="${HOME}/tmp/myPipeline"
export SRC_DIR="${WORK_DIR}/src"
export INSTALL_DIR="${WORK_DIR}/install"
export BUILD_DIR="${WORK_DIR}/build"
export GIT_URL="https://github.com/bioinfo-pf-curie/geniac-demo.git"

mkdir -p ${INSTALL_DIR} ${BUILD_DIR}

# clone the repository
# the option --recursive is needed if you use geniac as a submodule
git clone --recursive ${GIT_URL} ${SRC_DIR}


Let’s assume that the tool multiqc ( Ewels *et al*., 2016) must be added in the pipeline. The section params.geniac.tools of the file ${SRC_DIR}/conf/geniac.config must contain the following line:



multiqc = "conda-forge::lzstring=1.0.4=py_1001
       ↪  conda-forge::matplotlib-base=3.1.1=py37h250f245_2
       ↪  conda-forge::spectra=0.0.11=py_1 bioconda::multiqc=1.8=py_2"


The syntax follows the pattern from the conda package naming convention, so that the version is explicit as recommended in some guidelines ( Gruening *et al*., 2018):


toolName = "condaChannelName::softName=version=buildString"


If other Conda dependencies are required, they are added according to the same convention as shown for multiqc. In order to use the tool in a Nextflow process, use the label directive using the exact same name as given in the params.geniac.tools section. For example, the process multiqc in the file ${SRC_DIR}/main.nf contains the label multiqc defined in the file ${SRC_DIR}/conf/geniac.config:


process multiqc {
  label 'multiqc'
  
  // write your nextflow code.
  
}


### Add a Nextflow process for a tool available from source code

As not all the tools are available in Conda, we explain here how to add a new process in the pipeline when the tool is available from source code. It offers a great flexibility as the software developer can control the source code and tune the installation process. However, this obviously requires more configuration. In particular, the software developer has to be fluent with Cmake.

Let’s assume that the tool helloWorld must be added in the pipeline. Its source code must be copied in the folder ${SRC_DIR}/modules/fromSource/helloWorld. In order to use the tool in a Nextflow process, use the label directive using the exact same name as given to this folder (i.e. helloWorld). Then, the Cmake template script {SRC_DIR}/geniac/data/modules/fromSource/CMakeLists.txt is copied into {SRC_DIR}/modules/fromSource/CmakeLists.txt and the following Cmake directive is added:


ExternalProject_Add(

    helloWorld
    SOURCE_DIR ${CMAKE_CURRENT_SOURCE_DIR}/helloWorld
    CMAKE_ARGS
        -
DCMAKE_INSTALL_PREFIX=${CMAKE_BINARY_DIR}/externalProject/bin)


Lastly, the ${SRC_DIR}/modules/fromSource/helloWorld/CmakeLists.txt Cmake script must be written to detail how the tool must be compiled and installed, for example:


project(helloWorld LANGUAGES CXX)

project (helloWorld)

add_executable(helloWorld main.cpp)
install(TARGETS helloWorld DESTINATION ${CMAKE_INSTALL_PREFIX})


### Check the code with the linter

The Geniac command line interface is available in the conda environment which has been created and activated a the beginning of tthe *Use cases* section.

Run the linter on your repository:


geniac lint ${SRC_DIR}


### Build the Singularity containers

It consists of two main steps. First, the pipeline is configured with the required cmake options. The options with the pattern -DCMAKE_<UPPER_CASE>=<value> correspond to the variables available in the cmake language ( CMAKE_INSTALL_PREFIX defines where the analysis pipeline will be deployed). The options with the pattern -Dap_<lower_case>=<value> correspond to the *ad-hoc* variables defined by the Geniac toolbox (see the Geniac documentation for the list of available options). In this use case, the option -Dap_install_singularity_images=ON tells Geniac to build and install the Singularity images and -Dap_nf_executor=slurm to set SLURM as the executor (i.e. the job-scheduler) in the cluster profile used by Nextflow. Secondly, it starts the build of the pipeline with the make command. These two steps work as follows:



cd ${BUILD_DIR}

### configure the pipeline
cmake ${SRC_DIR}/geniac -DCMAKE_INSTALL_PREFIX=${INSTALL_DIR}
      ↪  -Dap_install_singularity_images=ON -Dap_nf_executor=slurm

### /!\ with sudo, the singularity and nextflow commands must be
### /!\ in the secure_path option declared in the file /etc/sudoers

### build the files needed by the pipeline
sudo make

### change file owner/group to the current user
sudo chown -R $(id -gn):$(id -gn) ${BUILD_DIR}


During the build process, Geniac generates the different configuration files, the Singularity recipes and images in the ${BUILD_DIR}/workDir folder as shown in Figure 3B. In particular, the ${BUILD_DIR}/workDir/results/conf/singularity.config file needed to run the singularity profile is created at this stage. Note that these files are not present in the ${SRC_DIR} as they are automatically generated. The root credentials are needed to build the Singularity images thus requiring the use of the sudo commands.

### Deploy the pipeline

The deployment of the pipeline is performed with the make command:



make install


After the deployment, the pipeline is available in the ${INSTALL_DIR}/pipeline folder in which the main.nf Nextflow file is available (see Figure 3C). Other folders are also created which contain the Singularity images, a folder (or a symlink depending on the option used during the configuration) for the genome annotations, and folders for the path and multipath profiles. In particular, the ${BUILD_DIR}/workDir/results/conf/singularity.config file created in the previous stage is deployed in the ${INSTALL_DIR}/conf/singularity.config file needed to run the singularity profile.

### Test and run the pipeline

As testing is generally a challenging part of the development and maintenance of the pipeline, we strongly recommend to create a conf/test.config profile along with a minimal dataset in the test/data folder such that it can be processed quickly to check that new developments did not introduce error. In this section, we show how to run the pipeline using this test profile.

The pipeline can be run with the singularity profile locally on the computer as follows:



cd ${INSTALL_DIR}/pipeline
nextflow -c conf/test.config run main.nf -profile singularity


The pipeline can be run with the singularity and cluster profiles on a high-performance computing cluster with the SLURM job-scheduler as follows:



nextflow -c conf/test.config run main.nf -profile singularity,cluster


Of note, running the pipeline with the test profile can be directly done from the ${BUILD_DIR} directory using the commands make test_singularity or make test_singularity_cluster respectively.

### Geniac command line interface

For users not familiar with cmake or make commands we propose a command line interface. The use case can be performed with the following commands:


export WORK_DIR="${HOME}/tmp/myPipeline_CLI"
export INSTALL_DIR="${WORK_DIR}/install"
geniac init -w ${WORK_DIR} ${GIT_URL}
cd ${WORK_DIR}
geniac lint
geniac install . ${INSTALL_DIR} -m singularity
sudo chown -R  $(id -gn):$(id -gn) build
geniac test singularity
geniac test singularity --check-cluster


### Compatibility with Nextflow DSL2

Geniac is fully compatible with Nextflow DSL2 provided that the process, workflows and subworkflows are organized in the ${SRC_DIR}/nf-modules folder as shown in red in Figure 3A. The Geniac Demo DSL2 ( La Rosa *et al*., 2022) repository can be used instead of the Geniac Demo . To do so just set, at the very beginning of the *Use cases* section, the value of GIT_URL to:


export GIT_URL="https://github.com/bioinfo-pf-curie/geniac-demo-dsl2.git"


## Conclusions

Geniac consists of a toolbox with three components: a technical **documentation** available at https://geniac.readthedocs.io , a **command line interface** with a linter to check that the code respects the guidelines, and an **add-on** to generate configuration files, build the container and deploy the pipeline. 

Compared to nf-core, Geniac builds upon their best-practises by adding additional guidelines, but is more flexible and can be easily adjusted to any pipeline template. More precisely, the only requirement in Geniac is the use of the label directive for each process which should use only one tool and one container at a time. The use of labels enables the *one container - one tool* strategy which was motivated by the development of Geniac . To do so, the specific conf/geniac.config file is added in the pipeline repository along with a specific profiles section (which differs from nf-core) in the nextflow.config to use Geniac .

The Geniac Demo and Geniac Demo DSL2 pipelines are available so that the reader can practice the Geniac guidelines. The Geniac Template makes it possible to start a new pipeline from scratch. The Geniac toolbox is fully compatible with Nextflow DSL1 and DSL2. While it supports the automatic generation of Docker and Singularity containers, it is straightforward to add any other containerization framework such as podman or shifter. The development of the Geniac toolbox was strongly motivated by the context of *production* analysis. However, we strongly encourage the use of Geniac in the context of the *research development* analysis. Our Bioinformatics Core facility uses the Geniac toolbox for a ten of pipelines deployed for *production* analysis covering a large variety of sequencing applications with both short-read and long-read technologies: it includes quality controls, analysis of ChIP-seq, ATAC-seq, RNA-seq, Whole Exome-seq, Whole Genome-seq and CRISPR data.

## Data availability

All data underlying the results are available as part of the article and no additional source data are required.

## Software availability

- Source code for geniac available from: https://github.com/bioinfo-pf-curie/geniac
- Archived source code at time of publication: https://doi.org/10.5281/zenodo.6039812 ( Hupé *et al*., 2022a)
- Source code for geniac-demo available from: https://github.com/bioinfo-pf-curie/geniac-demo
- Archived source code at time of publication: https://doi.org/10.5281/zenodo.6040173 ( Hupé *et al*., 2022b)
- Source code for geniac-demo-dsl2 available from: https://github.com/bioinfo-pf-curie/geniac-demo-dsl2
- Archived source code at time of publication: https://doi.org/10.5281/zenodo.6040166 ( La Rosa *et al*., 2022)
- Source code for geniac-template available from: https://github.com/bioinfo-pf-curie/geniac-template
- Archived source code at time of publication: https://doi.org/10.5281/zenodo.6040058 ( Servant & Hupé, 2022)
- All documentation is available at: https://geniac.readthedocs.io
- License: CeCILL Version 2.1

## References

[ref-17] da Veiga Leprevost F Barbosa VC Francisco EL : On best practices in the development of bioinformatics software. *Front Genet.* 2014;5:199. 10.3389/fgene.2014.00199 25071829 PMC4078907

[ref-13] Di Tommaso P Chatzou M Floden EW : Nextflow enables reproducible computational workflows. *Nat Biotechnol.* 2017;35(4):316–319. 10.1038/nbt.3820 28398311

[ref-23] Ewels P Magnusson M Lundin S : Multiqc: summarize analysis results for multiple tools and samples in a single report. *Bioinformatics.* 2016;32(19):3047–3048. 10.1093/bioinformatics/btw354 27312411 PMC5039924

[ref-19] Ewels PA Peltzer A Fillinger S : The nf-core framework for community-curated bioinformatics pipelines. *Nat Biotechnol.* 2020;38(3):276–278. 10.1038/s41587-020-0439-x 32055031

[ref-16] Georgeson P Syme A Sloggett C : Bionitio: demonstrating and facilitating best practices for bioinformatics command-line software. *Gigascience.* 2019;8(9):giz109. 10.1093/gigascience/giz109 31544213 PMC6755254

[ref-12] Goble C Cohen-Boulakia S Soiland-Reyes S : FAIR Computational Workflows. *Data Intell.* 2020;2(1–2):108–121. 10.1162/dint_a_00033

[ref-1] Goh WWB Wong L : The birth of bio-data science: Trends, expectations, and applications. *Genomics Proteomics Bioinformatics.* 2020;18(1):5—15. 10.1016/j.gpb.2020.01.002 32428604 PMC7393550

[ref-7] Gruening B Sallou O Moreno P : Recommendations for the packaging and containerizing of bioinformatics software [version 2; peer review: 2 approved, 1 approved with reservations]. *F1000Res.* 2018;7:ISCB Comm J-742. 10.12688/f1000research.15140.2 31543945 PMC6738188

[ref-8] Grüning B Dale R Sjödin A : Bioconda: sustainable and comprehensive software distribution for the life sciences. *Nat Methods.* 2018;15(7):475–476. 10.1038/s41592-018-0046-7 29967506 PMC11070151

[ref-20] Hupé P Allain F Roméjon J : bioinfo-pf-curie/geniac: version-2.0.0.2022a. 10.5281/zenodo.6039812

[ref-22] Hupé P Allain F Servant N : bioinfo-pf-curie/geniac-demo: version-2.0.0.2022b. 10.5281/zenodo.6040173

[ref-14] Jackson M Kavoussanakis K Wallace EWJ : Using prototyping to choose a bioinformatics workflow manage-ment system. *PLoS Comput Biol.* 2021;17(2):e1008622. 10.1371/journal.pcbi.1008622 33630841 PMC7906312

[ref-3] Jarlier F Joly N Fedy N : QUARTIC: QUick pArallel algoRithms for high-Throughput sequencIng data proCessing [version 3; peer review: 2 approved]. *F1000Res.* 2020;9:240. 10.12688/f1000research.22954.3 32913637 PMC7429925

[ref-15] Kamoun C Roméjon J de Soyres H : *biogitflow:* development workflow protocols for bioinformatics pipelines with git and gitlab. *F1000Res.* 2020;9:632. 10.12688/f1000research.24714.3 33732441 PMC7921891

[ref-10] Kurtzer GM Sochat V Bauer MW : Singularity: Scientific containers for mobility of compute. *PLoS One.* 2017;12(5):e0177459. 10.1371/journal.pone.0177459 28494014 PMC5426675

[ref-24] La Rosa P Hupé P Roméjon J : bioinfo-pf-curie/geniac-demo-dsl2: version-2.0.0.2022. 10.5281/zenodo.6040166

[ref-18] Lawlor B Walsh P : Engineering bioinformatics: building reliability, performance and productivity into bioinformatics software. *Bioengineered.* 2015;6(4):193–203. 10.1080/21655979.2015.1050162 25996054 PMC4601517

[ref-4] Leipzig J : A review of bioinformatic pipeline frameworks. *Brief Bioinform.* 2017;18(3):530–536. 10.1093/bib/bbw020 27013646 PMC5429012

[ref-9] Merkel D : Docker: Lightweight linux containers for consistent development and deployment. *Linux J.* 2014;2014(239). Reference Source

[ref-2] Reiter T Brooks PT Irber L : Streamlining data-intensive biology with workflow systems. *Gigascience.* 2021;10(1):giaa140. 10.1093/gigascience/giaa140 33438730 PMC8631065

[ref-21] Servant N Hupé P : bioinfo-pf-curie/geniac-template: version-2.0.0.2022. 10.5281/zenodo.6040058

[ref-5] Strozzi F Janssen R Wurmus R : Scalable Workflows and Reproducible Data Analysis for Genomics. *Methods Mol Biol.* 2019;1910:723–745. 10.1007/978-1-4939-9074-0_24 31278683 PMC7613310

[ref-6] Tanjo T Kawai Y Tokunaga K : Practical guide for managing large-scale human genome data in research. *J Hum Genet.* 2021;66(1):39–52. 10.1038/s10038-020-00862-1 33097812 PMC7728600

[ref-11] Wilkinson MD Dumontier M Aalbersberg IJJ : The fair guiding principles for scientiﬁc data management and stewardship. *Sci Data.* 2016;3:160018. 10.1038/sdata.2016.18 26978244 PMC4792175

