# Supplementary material for: Geniac: Automatic Configuration GENerator and Installer for nextflow pipelines
Source: Open Res Eur. 2022 Feb 21;1:76. Originally published 2021 Jul 2. [Version 2] doi: 10.12688/openreseurope.13861.2 (PMC10445886; doi:10.12688/openreseurope.13861.2)
Supplement: Peer review history

# Peer review: Reviewer response for version 2

**DOI:** 10.21956/openreseurope.15693.r28633

**Published:** 2022-03-24

**Authors:** Frédéric Lemoine

**Affiliations:** 1 Evolutionary Genomics of RNA viruses & Bioinformatics and Biostatistics Hub, F-75015, Institut Pasteur, Université Paris Cité, Paris, France

I thank the authors for the work they did to address the points I raised and to make the manuscript clearer.

 I have no further comments to make.

Is the rationale for developing the new software tool clearly explained?

Yes

Is the description of the software tool technically sound?

Partly

Are sufficient details of the code, methods and analysis (if applicable) provided to allow replication of the software development and its use by others?

Partly

Is sufficient information provided to allow interpretation of the expected output datasets and any results generated using the tool?

Yes

Reviewer Expertise:

I work in the field of evolutionary bioinformatics where I develop tools and workflows. I am interested in the reproducibility of bioinformatics analyses, and I use Nextflow and container technologies on a daily basis.

I confirm that I have read this submission and believe that I have an appropriate level of expertise to confirm that it is of an acceptable scientific standard.

# Peer review: Reviewer response for version 1

**DOI:** 10.21956/openreseurope.14944.r27224

**Published:** 2021-08-31

**Authors:** Frédéric Lemoine

**Affiliations:** 1 Evolutionary Genomics of RNA viruses & Bioinformatics and Biostatistics Hub, F-75015, Institut Pasteur, Université Paris Cité, Paris, France

This manuscript describes Geniac, a toolbox aiming at increasing the reproducibility and reusability of Nextflow workflows. To do so, Geniac has the following features:

1. It defines a set of good practices for the development of Nextflow workflows, via a detailed documentation.
2. It provides a linter to check that the best coding practices are respected.
3. It provides a "add-on" that automatically builds dependencies, generates the singularity (or docker) containers needed to run the workflow, checks the configuration files, and installs the workflow in the specified directory ("deploy").

Geniac tackles an important issue with bioinformatic analyses: Pipelines are rarely homogeneous in terms of description and implementation, which leads to reproducibility, maintenance, and reusability difficulties. In terms of goals, Geniac present some similarities with nf-core, which aims at curating and harmonizing pipeline implementations.The solution proposed by Geniac is to define of a set of homogeneous practices for workflow development (e.g. how to describe process dependencies, how to name variables, channels and files, and provide tools to check workflows, build containers and install the workflow). The price to pay for more reproducible and homogeneous workflows is a more complex technological stack (with CMake specifically) and workflow architecture. Technically, Geniac is based on the following technologies: Nextflow, conda, Docker (or Singularity) and CMake. Although technical, the manuscript explains clearly the philosophy, the goals, the usage and the usefulness of Geniac. The tools are usable in the provided workflow example, I was able to run the geniac-demo and the linter works well and is easy to use.

 I believe that Geniac is a useful tool that answers important questions. However, I think it would be easier to understand and use with a few clarifications in my opinion:

- I had some initial difficulties to run the geniac-demo workflow. I think the documentation (on readthedoc and on the git repositories) could be clearer about how to run the workflow in different situations, at least with docker or singularity. For example, I initially had an error when the install and the build directories were included in the geniac-demo directory. Before understanding the general philosophy of Geniac, I also had some difficulties to understand how to generate the singularity.config file needed to generate singularity images and run the workflow. A more visible step by step concrete quick-start example could be very useful.
- Following the previous point, I was a bit confused by the README in the geniac-demo and geniac-template repositories, where I was looking for information on how to run the example. The documentation could be better adapted to the workflows. For example the geniac.md file refers to nf-CRISPR repository.
- Testing the workflow is also generally a difficult part of the development and maintenance of workflows. It is not mentioned clearly in the introduction or in the discussion, though I think that tests can be performed using test profiles in Geniac. I think it would be great to give a little bit more insights on what tests consist of in geniac workflows.
- The abstract states that workflow systems + containers increase usage complexity. I am not sure that Geniac tackles this problem, since it adds a new layer of complexity in the top of Nextflow, Docker, etc (which is unavoidable). If I understand correctly, Geniac is mainly solving the problem of workflow homogeneity in terms of structure, description, and documentation, and thus make the workflow more reproducible, maintainable and reusable.
- A more detailed description of the specificities of Geniac compared to nf-core may be a great addition to the manuscript.
- Are there already any public real-world Geniac compatible workflows in production? If so, it could be interesting to mention a few of them.

A few typos/suggestions :   

- Abstract+Introduction: "which address" => "which target"?
- Introduction: "the workflow has no choice but deliver": Isn't it more the facility rather than the workflow that has no choice?
- Introduction: "being less invasive to the different expert communities": This point is not very clear to me. Would it be possible to rephrase it?
- Methods: "to be as granular as possible", maybe clearer with "to obtain the finest level of granularity"?
- Methods: "The building of the containers can be parallelized to reduce the time in which...". It is not very clear to me. Would something like "to speed up the deployment of new containers" make sense?
- The DOI link in the ref Servant & Hupé 2021 does not work (there is an additional space in the URL).
-  Methods: "Other folders are also created which contains"=>"contain".

Is the rationale for developing the new software tool clearly explained?

Yes

Is the description of the software tool technically sound?

Partly

Are sufficient details of the code, methods and analysis (if applicable) provided to allow replication of the software development and its use by others?

Partly

Is sufficient information provided to allow interpretation of the expected output datasets and any results generated using the tool?

Yes

Reviewer Expertise:

I work in the field of evolutionary bioinformatics where I develop tools and workflows. I am interested in the reproducibility of bioinformatics analyses, and I use Nextflow and container technologies on a daily basis.

I confirm that I have read this submission and believe that I have an appropriate level of expertise to confirm that it is of an acceptable scientific standard, however I have significant reservations, as outlined above.

# Response

**Published:** 2022-02-11

**Authors:** Philippe HUPE

First, we would like to thank the reviewer. We are very grateful for his time, contribution and very valuable comments which significantly helped to improve the article and the documentation of the Geniac. You will find below a detailed answer to the different issues that we have addressed in the revised manuscript. Best regards, Philippe Hupé. (Reviewer comments in italics)

*I had some initial difficulties to run the geniac-demo workflow. I think the documentation (on readthedoc and on the git repositories) could be clearer about how to run the workflow in different situations, at least with docker or singularity. For example, I initially had an error when the install and the build directories were included in the geniac-demo directory. Before understanding the general philosophy of Geniac, I also had some difficulties to understand how to generate the singularity.config file needed to generate singularity images and run the workflow. A more visible step-by-step concrete quick-start example could be very useful.*

*Following the previous point, I was a bit confused by the README in the geniac-demo and geniac-template repositories, where I was looking for information on how to run the example. The documentation could be better adapted to the workflows. For example the geniac.md file refers to nf-CRISPR repository*.

**Reply: **We are sorry that the geniac-demo was not relevant as it was simply a copy/paste from the geniac-template. Therefore, the geniac-demo documentation has been modified. As suggested by the reviewer1, we included in the README a "Quick start" section such that the reader can test and run the pipeline either with the multiconda or the singularity profile using the new command line interface.

       

*Testing the workflow is also generally a difficult part of the development and maintenance of workflows. It is not mentioned clearly in the introduction or in the discussion, though I think that tests can be performed using test profiles in Geniac. I think it would be great to give a little bit more insights on what tests consist of in geniac workflows*.

**Reply**: Testing is indeed a major component of software development. Therefore, the section "Run the pipeline" has been renamed "Test and run the pipeline". We highlighted the necessity to include a test profile and test data. We have also indicated that geniac integrates several Make commands to facilitate the test of the pipeline.

       

*The abstract states that workflow systems + containers increase usage complexity. I am not sure that Geniac tackles this problem, since it adds a new layer of complexity in the top of Nextflow, Docker, etc (which is unavoidable). If I understand correctly, Geniac is mainly solving the problem of workflow homogeneity in terms of structure, description, and documentation, and thus make the workflow more reproducible, maintainable and reusable.*

**Reply**: The abstract has been modified as suggested to mention that "promoting the homogeneity of the workflow implementation requires guidelines and protocols" such as Geniac intends.

       

*A more detailed description of the specificities of Geniac compared to nf-core may be a great addition to the manuscript*.

**Reply:** We added a new paragraph in the Conclusion to explain how Geniac compares to nf-core.

*Are there already any public real-world Geniac compatible workflows in production? If so, it could be interesting to mention a few of them. Indeed, geniac is used in our Bioinformatics Core Facility.*

**Reply:** This has now been added in the Conclusion.

A few typos/suggestions :  > * Abstract+Introduction: *"which address" => "which target"?*

**Reply: **This has been corrected.

    

 I *ntroduction: "the workflow has no choice but deliver": Isn't it more the facility rather than the workflow that has no choice?*

**Reply: **This is right, the sentence has been rephrased.

*Introduction: "being less invasive to the different expert communities": This point is not very clear to me. Would it be possible to rephrase it?*

**Reply:** This has been rephrased to explain that geniac wants to preserved the preferred work habits of the developers.

    

*Methods: "to be as granular as possible", maybe clearer with "to obtain the finest level of granularity"?*

**Reply: **We have replaced granular by modular since the idea of "one container - one tool" strategy is that each container can be seen as a module which performs a given task.

*Methods: "The building of the containers can be parallelized to reduce the time in which...". It is not very clear to me. Would something like "to speed up the deployment of new containers" make sense?*

**Reply**: Indeed, the sentence has been rephrased as suggested.

*The DOI link in the ref Servant & Hupé 2021 does not work (there is an additional space in the URL).*

**Reply: **This has been corrected.

    

*Methods: "Other folders are also created which contains"=>"contain".*

**Reply: **This has been corrected.

# Peer review: Reviewer response for version 1

**DOI:** 10.21956/openreseurope.14944.r27225

**Published:** 2021-08-02

**Authors:** Toni Hermoso Pulido

**Affiliations:** 1 Centre for Genomic Regulation, The Barcelona Institute of Science and Technology, Barcelona, Spain

In this article, the Geniac toolbox is presented. The software consists of several tools to address some common difficulties of deploying and adapting Nextflow pipelines to fit into different computing environments. Its major emphasis is ensuring both code reproducibility and production-level reliability. Technically speaking, it depends on Nextflow itself and CMake automation and packaging tool. A Python-based code analysis (linter) tool is also provided for checking pipeline setup in advance before the actual build and deployment process takes place.

 Provided software for the pipelines relies primarily on Conda package manager, which Geniac facilitates to adapt to different approaches (e.g., single or multi), and converts to handy container images such as Singularity-based ones.

 Experienced practitioners familiar with Nextflow, Conda and container technologies can easily follow the instructions and customize the provided examples (which are based on both legacy and DSL2 Nextflow syntax). Maybe the final result and associated folders of the pipeline preparation (build, install), as depicted in Figure 3, could be more clearly stated.

 Despite it being mentioned in the article and in the toolbox documentation as an actual possibility, a more explicit example case of a software step that does not rely on Conda could be a nice addition.

 As a user experience suggestion for future releases, it could be interesting that the *geniac* executable could be already provided straight from the Python Package Index (PyPI). It would also help new adopters, specially if not so familiar with CMake, if *geniac* could wrap some of the execution calls that are included in the useCases.bash script.

Is the rationale for developing the new software tool clearly explained?

Yes

Is the description of the software tool technically sound?

Yes

Are sufficient details of the code, methods and analysis (if applicable) provided to allow replication of the software development and its use by others?

Yes

Is sufficient information provided to allow interpretation of the expected output datasets and any results generated using the tool?

Partly

Reviewer Expertise:

I work in Bioinformatics. I have experience developing and deploying Nextflow pipelines, and I regularly take care of infrastructure and devops-related tasks.

I confirm that I have read this submission and believe that I have an appropriate level of expertise to confirm that it is of an acceptable scientific standard.

# Response

**Published:** 2022-02-11

**Authors:** Philippe HUPE

First, we would like to thank the reviewer. We are very grateful for his time, contribution and very valuable comments which significantly helped to improve the article and the documentation of the Geniac. You will find below a detailed answer to the different issues that we have addressed in the revised manuscript. Best regards, Philippe Hupé. (Reviewer comments in italics)

*In this article, the Geniac toolbox is presented. The software consists of several tools to address some common difficulties of deploying and adapting Nextflow pipelines to fit into different computing environments. Its major emphasis is ensuring both code reproducibility and production-level reliability. Technically speaking, it depends on Nextflow itself and CMake automation and packaging tool. A Python-based code analysis (linter) tool is also provided for checking pipeline setup in advance before the actual build and deployment process takes place.*

*Provided software for the pipelines relies primarily on Conda package manager, which Geniac facilitates to adapt to different approaches (e.g., single or multi), and converts to handy container images such as Singularity-based ones.*

*Experienced practitioners familiar with Nextflow, Conda and container technologies can easily follow the instructions and customize the provided examples (which are based on both legacy and DSL2 Nextflow syntax). Maybe the final result and associated folders of the pipeline preparation (build, install), as depicted in Figure 3, could be more clearly stated.*

**Reply:** The Figure 3B and 3C have been modified to highlight where the files automatically generated in the build directory are copied in the install directory.

*Despite it being mentioned in the article and in the toolbox documentation as an actual possibility, a more explicit example case of a software step that does not rely on Conda could be a nice addition.*

**Reply:** We added a new section in the manuscript to describe how to "Add a Nextflow process for a tool available from source code".

*As a user experience suggestion for future releases, it could be interesting that the geniac executable could be already provided straight from the Python Package Index (PyPI).*

**Reply: **The Geniac command line interface is now available on PyPI at https://pypi.org/project/geniac/.

*It would also help new adopters, specially if not so familiar with CMake, if geniac could wrap some of the execution calls that are included in the useCases.bash script.*

**Reply:** New options have been added to the Geniac command line interface to facilitate the use of geniac for developers not familiar with CMake commands. The new section "Geniac command line interface" has been added in the manuscript to illustrate what command lines can be used for the proposed use case. The documentation has been updated with these new functionalities.
